# *In Vitro* and *In Vivo* Antitumor Activity of *Scutellaria barbate* Extract on Murine Liver Cancer

**DOI:** 10.3390/molecules16064389

**Published:** 2011-05-27

**Authors:** Zhi-Jun Dai, Jie Gao, Zong-Fang Li, Zong-Zheng Ji, Hua-Feng Kang, Hai-Tao Guan, Yan Diao, Bao-Feng Wang, Xi-Jing Wang

**Affiliations:** 1 Department of Oncology, the Second Affiliated Hospital, Medical School of Xi’an Jiaotong University, Xi’an 710004, China; 2 Department of General Surgery, the Second Affiliated Hospital, Medical School of Xi’an Jiaotong University, Xi’an 710004, China

**Keywords:** anti-tumor, *Scutellaria barbate*, hepatoma, immune function, mice

## Abstract

In the present study, we investigated the *in vitro* and *in vivo* antitumor effects of crude extract of *Scutellaria barbate* (CE-SB) on mouse hepatoma H22 cells. The MTT assay was used to determine the growth inhibition of H22 cells *in vitro*. The *in vivo* therapeutic effects of CE-SB were determined using H22 tumor bearing mice. Besides, the body weight, tumor weight, thymus index and spleen index of H22 bearing mice were also measured. The tumor inhibitory rate (IR) was calculated according to the mean weight of tumor (MWT). The phagocytotic function of macrophages was examined by observing peritoneal macrophages phagocytize chicken RBC. The results showed that CE-SB could inhibit the growth of hepatoma H22 Cells *in vitro* and *in vivo*. Furthermore, CE-SB could improve immune function of H22 tumor bearing mice. Together these results indicate that CE-SB has antitumor activity and seems to be safe and effective for the use of anti-tumor therapy.

## 1. Introduction

Liver cancer is a malignant diseases with a high incidence and mortality which is often diagnosed at an advanced stage [1]. As is known to all, the common therapies such as resection, transplantation or percutaneous and transarterial interventions are of limited efficacy [2]. Currently, chemotherapy is one of the important treatment methods, but the side effects are difficult to tolerate, and as a result, people have being paying more attention to searching for new antitumor agents from natural products [3]. Many Chinese herbs have been discovered to be potential sources of antitumor drugs [4]. 

**Figure 1:**
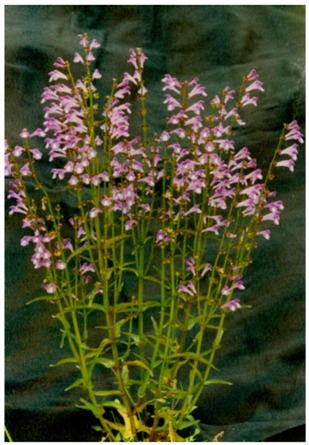
Photograph of *Scutellaria barbata* D. Don.

*Scutellaria barbata* D. Don (Figure 1) is a perennial herb, which mainly grows throughout southern China. This herb, known in traditional Chinese Medicine as Ban-Zhi-Lian, has been used as an anti-inflammatory and antitumor agent and also a diuretic in China and Korea [5,6,7,8,9,10,11,12,13,14]. Previous studies have reported that *Scutellaria barbata* D. Don contains a large number of alkaloids, flavones, steroids, and polysaccharides [15,16,17,18,19]. Recently, a variety of alkaloids has been isolated from *Scutellaria barbata* D. Don [16,17,18,19]. The new isolated compounds showed cytotoxic activities against many human cancer lines (HONE-1 nasopharyngeal, KB oral epidermoid carcinoma, and HT29 colorectal carcinoma cells) *in vitro* [16,17,18]. However, the active site of chemical structure for antitumor activity has not been fully determined [20]. In the clinic, this herb has been used in the treatment of lung cancer, digestive system cancers, hepatoma, breast cancer, and chorioepithelioma. Extracts of *Scutellaria barbata* D. Don (E-SB) have been shown to have *in vitro* growth inhibitory effects on a number of human cancers including leukemia, colon cancer, hepatoma and skin cancer [8,9,10,11,12,13,14]. However, further research is needed to investigate the antitumour effect and its mechanisms. The antitumor activity of crude extract of SB (CE-SB) was studied *in vitro* and *in vivo*.

## 2. Results and Discussion

### 2.1. CE-SB Inhibited the Proliferation of H22 Cells *In Vitro*

**Figure 2:**
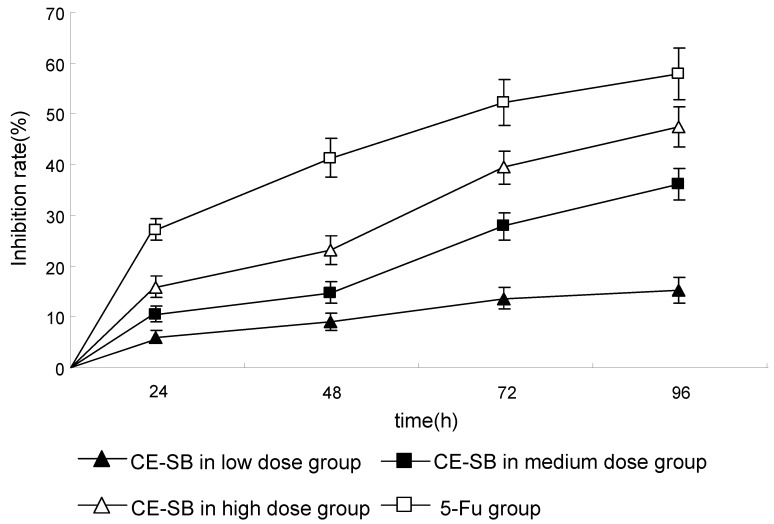
Growth inhibiting effects of CE-SB on hepatoma H22 cells. H22 cells were treated with low-high dose of CE-SB for 96 h. Dose- and time-dependent inhibition of H22 cell growth could be observed (*P* < 0.05, ANOVA analysis). Cells were incubated with different dosages of CE-SB and cell proliferation was determined by MTT assay.

The anti-proliferative effect of CE-SB on H22 cells was examined by MTT assays. Cells were treated with medium and different doses of CE-SB, and the inhibition rate was evaluated after 0, 24, 48, 72, and 96 hours. The results showed CE-SB in high dose and medium dose groups could inhibit the proliferation of H22 cells, while the inhibition effect of CE-SB in low dose group was not obvious. As shown in Figure 2, the inhibitory rate of CE-SB on cell growth is as high as (36.3 ± 4.5)% and (47.5 ± 6.4)%, when the cells are treated for 96 hours with medium and different doses of CE-SB. CE-SB inhibited the proliferation of H22 cells in a dose- and time-dependent manner.

In this study, we investigated the effects of ESB on inducing apoptosis of H22 cells with serum pharmacology. Serum pharmacology, which was put forward by Tashino [21], is divided into three steps: animal administration, serum collection and isolation, and pharmacological experiments on drug-containing serum. This method matches the effects of drugs with the *in vivo* pharmacological process, which is applicable to Chinese medicine, especially for efficacy evaluation of its mechanism of action of a compound. Many researchers believe that Serum Pharmacology is more scientific and more befitting for Chinese traditional medicine than traditional pharmacology in which crude drugs are directly added into the culture system of cells or organs *in vitro* [22,23,24].

### 2.2. Ultrastructure Observation of H22 Cells Induced by CE-SB

**Figure 3:**
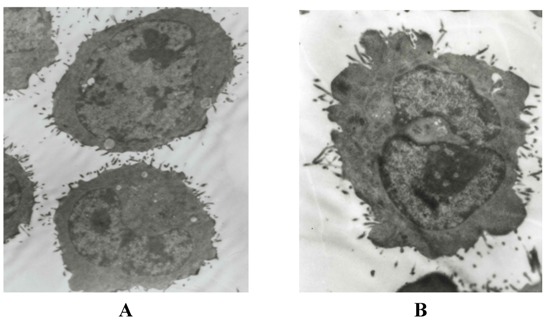
Morphological observation of H22 cells by EM after treatment. The cells were examined under a transmission electronmicroscope (×5000 power, bar = 1 μm). **A**: normal hepatoma H22 cells; **B**: karyopyknosis, membrane integrity and formation of apoptotic bodies in high dosage CE-SB group.

Through a high resolution transmission electron microscopy, normal H22 cells were round and regular, the chromatin margination showed in few tumor cells (Figure 3A). After treatment with high dose CE-SB for 48 h, part of nuclear membrane domed outward with a sharp angle. The typical morphology of apoptotic H22 cells such as chromatic agglutination and fragmentation of nuclei, chondriosome swelling, formation of apoptotic body could be observed in CE-SB high dose group (Figure 3B).

### 2.3. Anti-Tumor Effects of CE-SB on Hepatoma H22 Bearing Mice

All the mice inoculated with H22 cells were successively transplanted with liver cancer. Mice in the model control group were not lively, with dark fur, loss of appetite and slow weight gain; mice in the 5-FU group even died before the end of injection; in contrast, mice in the CE-SB group were vigorous with shiny fur and rapid body weight increases. The toxicity in the 5-FU group was serious and the concrete manifestations included anorexia, abdominal distention and athrepsy, as well as decrease in body mass, while the mice in CE-SB groups showed no evident toxicity.

As shown in Table 1, compared with the NS group, the tumors of the CE-SB and 5-FU groups shrank significantly (*P* < 0.01), while the tumor weight of three CE-SB groups had no statistical difference when compared with the 5-FU group (*P* > 0.05). The tumor inhibitory rates of the 5-FU group and CE-SB groups were 42.26%, 14.34%, 28.68% and 36.98%, respectively. There was no significant difference found in body weight between the CE-SB groups and the tumor control group before treatment (*P* > 0.05). After experiment, there was no significant difference found in the variation of body weight between the CE-SB groups and the tumor control group (*P* > 0.05). Compared with the tumor control groups, the weight of mice in the 5-FU group decreased significantly (*P* < 0.05).

The antitumor effect might be related to the components such as diterpenoid alkaloids [16,17,18,19], and flovonoids [25,26] in CE-SB. In this study, the whole plant extract was used. The concrete mechanism and links remains a topic for further study.

**Table 1:** Effects of CE-SB on body weight and tumor weight of H22 bearing mice ( 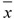 ± s, n = 10).

Group	Dosage (g/kg/d)	Body weight(g)		Variation of body weight (g)	Tumor weight (g)	Tumor inhibition rate (%)
		Pre-treatment Post-treatment				
Normal	－	20.03 ± 1.94	24.12 ± 2.02 ^#^	4.09 ± 2.32 ^#^	－	－
Tumor control	－	20.03 ± 2.07	23.50 ± 3.24 ^#^	3.47 ± 3.82 ^#^	2.65 ± 1.12 ^#^	－
5-FU	0.02	19.96 ± 2.03	20.74 ± 1.86 ^*^	0.78 ± 1.93 ^*^	1.53 ± 0.64 ^*^	42.26
CE-SB	3	20.00 ± 1.94	24.52 ± 1.82 ^#^	4.52 ± 3.93 ^#^	2.27 ± 0.93 ^#^	14.34
	6	19.93 ± 2.12	24.77 ± 1.98 ^#^	4.84 ± 3.62 ^#*^	1.89 ± 0.82 ^*#^	28.68
	12	20.01 ± 2.10	25.15 ± 2.13 ^#^	5.14 ± 3.76 ^*#^	1.67 ± 0.76 ^*^	36.98

^*^ *P* < 0.05 *vs.* tumor control group; ^#^ *P* < 0.05 *vs.* 5-FU group

### 2.4. Influence of CE-SB on Immune Organs and WBC Count of H22 Bearing Mice

**Figure 4:**
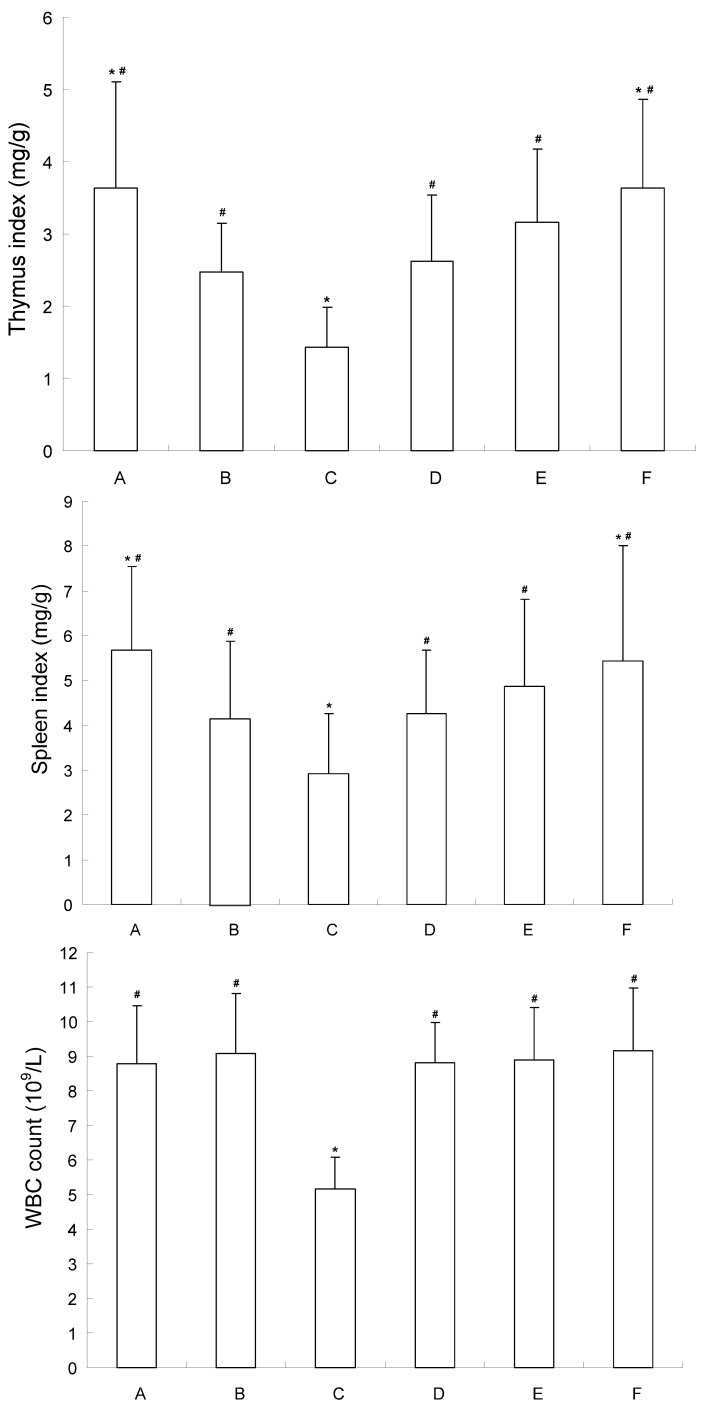
Influence of CE-SB on immune organs and WBC count of H22 bearing mice. Group A: normal control; Group B: tumor control; Group C: 5-FU; Group D: low dosage CE-SB; Group E: medium dosage CE-SB; Group F: high dosage CE-SB. * *P* < 0.05 *vs*. tumor control group; ^#^ *P* < 0.05 *vs*. 5-FU group.

As shown in Figure 4, compared with the tumor control group, the thymus index and spleen index of mice in the 5-FU group decreased significantly (*P* < 0.05). However, there was no significant difference between the low-medium dosage CE-SB group and the tumor control group. These results indicated that CE-SB did not inhibit the functions of immune organs. The WBC count was obviously decreased in 5-FU group, while it showed no change in the CE-SB groups, suggesting that CE-SB caused no bone marrow suppression.

### 2.5. Influence of CE-SB on Phagocytotic Function of Macrophage of H22 Bearing Mice

**Figure 5:**
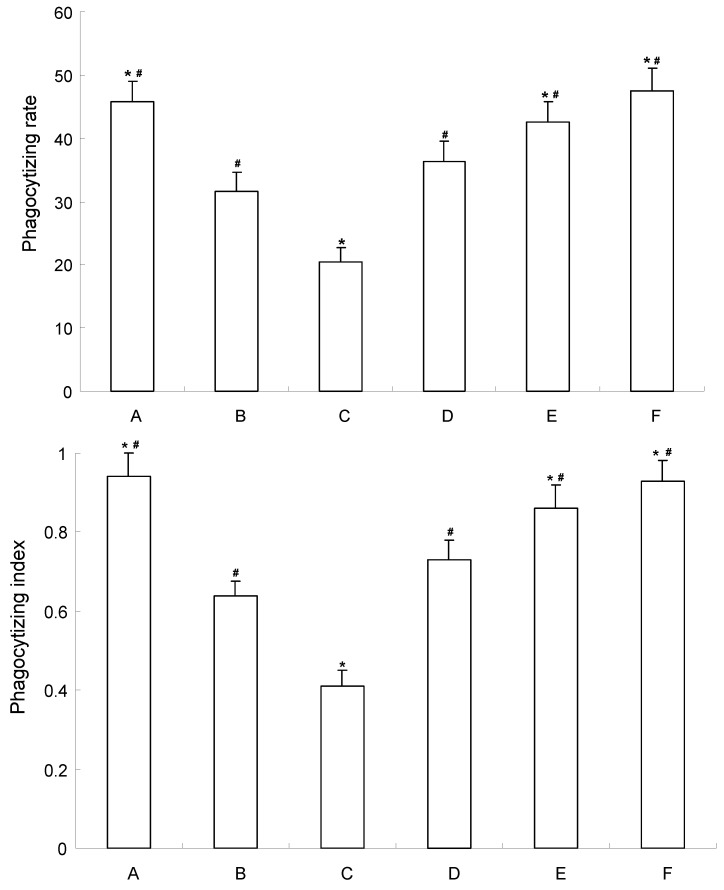
Influence of CE-SB on phagocytotic function of macrophage of H22 bearing mice. Group A: normal control; Group B: tumor control; Group C: 5-FU; Group D: low dosage CE-SB; Group E: medium dosage CE-SB; Group F: high dosage CE-SB. * *P* < 0.05 *vs.* tumor control group; ^#^ *P* < 0.05 *vs.* 5-FU group.

As shown in Figure 5, compared with the tumor control group, 5-FU decreased the phagocytic index and phagocytic rate significantly (*P* < 0.05). However, the phagocytotic function of macrophages of mice were increased in medium-high dosage CE-SB groups. The phagocytotic rate of medium dosage CE-SB group and high dosage CE-SB group were increased 34.92% and 55.75%, respectively, compred to the tumor control group. The phagocytotic indexes of the medium dosage CE-SB group and high dosage CE-SB group were increased 29.36% and 48.52%, respectively, compared to the tumor control group.

The phagocytotic function of macrophages can reflect the immune function to some extent [27]. The results suggest that CE-SB could enhance the immune function of H22-bearing mice. It is possible to estimate SB could improve the quality of life of patients suffering with malignant disease under clinical conditions. This effect might be related to the components such as steroids and polycose [28], but the mechanism of action needs further study to confirm this.

## 3. Experimental

### 3.1. Reagents and Animals

Murine H22 hepatoma cell was purchased from the Shanghai Institute of Cell Biology at the Chinese Academy of Sciences (Shanghai, P.R. China). RPMI1640 medium (Gibco, USA); Fetal bovine serum (Gibco, USA); Dimethyl sulfoxide (DMSO) and 3-(4,5-dimethylthiazol-2-yl)-2,5- diphenyltetrazolium bromide (MTT) were purchased from Sigma Chemical (St. Louis, MO, USA). Rats (SD rats, male, 220–250 g) and mice (ICR mice, half male and half female, 20 ± 2 g) were purchased from the Experiment Animal Center, Medical School of Xi'an Jiaotong University, Xi’an, P.R. China.

### 3.2. Preparation of *S. barbata* Extract and Drug Containing Serum

*S. barbata* crude extract (CE-SB) was purchased from Xi’an zhong-xin Biotechnology Development Ltd. (Xi’an, P.R. China). CE-SB-containing serum was prepared as previously described [29]. Twenty male SD rats were randomly divided into control group, high CE-SB dose group, medium CE-SB dose group, and low CE-SB dose group (n = 5). Rats in the high, medium and low CE-SB dose groups received intragastric CE-SB of 6, 3 and 1.5 g/d per kg of body weight. Rats in the control group received normal saline, twice a day for 3 d. Two hours after the last administration, blood was immediately obtained from the heart and kept at room temperature for 4 h. The serum was separated by centrifugation at 2,400 × g for 10 min, collected following twice of filtration with a 0.22 μm cellulose acetate membrane, calefied in 56 °C water for 30 min, and stored at −20 °C for use.

### 3.3. Cell Culture and Cell Proliferation Assay

Cells were cultured in RPMI-1640 medium supplemented with 10% fetal bovine serum, 1 × 10^5^ U/L penicillin and 100 mg/L streptomycin in a humidified atmosphere with 5% CO2 incubator at 37 °C. The cells were subcultured until reaching logarithmic growth phase. 

The MTT assay was used to determine the effect of CE-SB on the proliferation of H22 cells. H22 cells were seeded at a concentration of 5 × 10^3^ cell /well in 96-well plate, and grown at 37 °C, 5% CO_2_ incubator until adherence. After an overnight incubation in starvation medium containing 0.5% FBS, the cells on the culture plate were divided into groups on the basis of parallel lines, each group had four wells in one line for each group. At the end of the treatment, MTT (5 mg/mL, 20 μL) was added and the cells were incubated for another 4 hours. DMSO (200 μL) was added to each well after removing the supernatant. After shaking the plate for 10 mins. in the shaking board, cell viability was obtained by measuring the absorbance at 490 nm wavelength using Enzyme-labeling instrument (EX-800 type), this assay was done quintuplicate. The inhibition rate was calculated using the following formula:

Inhibition rate(%) = [1-(average absorbance of experimental group/ average absorbance of blank control group)] × 100%

### 3.4. Ultrastructure Observation by Transmission Electron Microscope

Staining of cells with uranyl acetate and lead citrate was performed to detect morphological changes. Briefly, adherent H22 cells were treated with CE-SB in high dose for 48 hours. After treatment, the treated cells were digested with pancreatin and fixed with 3% glutaraldehyde precooled in 4 °C for 2 h. To make ultra-thin sections of copper, cells were washed with PBS once, fixed with 1% osmic acid for one more hour, dehydrated by acetone and embedded by epoxide resin. After stained with uranyl acetate and lead citrate, the sections were examined by a Hitachi-800 transmission electron microscope as previously described [30].

### 3.5. Antitumor Activity *In Vivo*

ICR mice were divided into six groups (n = 20). All the animals were injected with H22 cells (2 × 10^6^ cells /mouse) into the armpit of right hind limb subcutaneously, except for the normal group. This was taken as day zero. Group A served as the normal control and group B served as the tumor control. These two groups received sodium suspension (0.9%). Group C, which served as the positive control, was treated with the suspension of 5-FU at 20 mg/kg body weight. Groups D, E, and F were treated with CE-SB at 3, 6, 12 g/d per kg of body weight, respectively. All these treatments were given 24 h after the tumor inoculation, once daily for 12 days.

After the last dose and 24 h fasting, 10 mice from each group were sacrificed. The rest 10 mice were kept to observe survival time for five weeks. The weight of tumor mass and mouse were examined. The anti-tumor activity was evaluated by tumor weighing. The following formula was used:

Tumor inhibitory rate (%) = (1-average tumor weighing of administration group/ average tumor weighing of the control) × 100% [31].

The blood was collected from the animals by retro-orbital puncture under slight anesthesia (diethyl ether) condition; and the hematological parameters such as red blood cells (RBC), white blood cells (WBC) and hemoglobin content were determined. At the same time, thymus and spleen were taken from mice. The impact on immune organ was evaluated based on the thymus index and spleen index [32].

### 3.6. Determination of Phagocytotic Function of Macrophage

After the last dose and 24 h fasting, chicken-red cells (1 mL, 5%) were injected intraperitoneally into each group. After 12 h, the mice were sacrificed and normal saline (2 mL) was injected into the abdominal cavity. Then peritoneal fluid (1 mL) was drawn for glass slides. After incubating for 30 min, peritoneal fluid was fixed with a mixture of acetone/methanol (1:1, v/v) and stained with 4% Giemsa. After drying, peritoneal macrophages were counted under microscope. The effect of CE-SB on phagocytosis of enterocoelia macrophage was evaluated by the chicken-red cell phagocytic rate and phagocytic index [33], which were calculated using the following formulas:

Phagocytotic rate = (macrophages which phagocyted RBC/ total macrophages) × 100%;

Phagocytotic index = chicken-red cells phagocyted / total macrophages.

### 3.7. Statistical Analysis

All values were expressed as the mean ± SEM. Statistical analysis was performed with one-way analysis of variance(ANOVA) and student *t* test using the statistical software SPSS 11.0. *P* < 0.05 was considered as statistically significant. 

## 4. Conclusions

In conclusion, the CE-SB was observed to inhibit the proliferation of H22 cells *in vitro* in a dose and time dependent manner. CE-SB could inhibit the growth of H22 implanted tumor and enhance the immune function of H22 tumor-bearing mice *in vivo*. These results indicated that CE-SB had antitumor activity, besides, it might be safe and effective for the use in anti-tumor therapy. However, further studies are necessary to clarify the detailed mechanism(s) involved in the observed antitumor effects of CE-SB.
